# From Microbes to Myocardium: A Comprehensive Review of the Impact of the Gut-Brain Axis on Cardiovascular Disease

**DOI:** 10.7759/cureus.70877

**Published:** 2024-10-05

**Authors:** Akhilesh Singh, Prem S Kishore, Sharleen Khan

**Affiliations:** 1 Emergency Medicine, Jawaharlal Nehru Medical College, Datta Meghe Institute of Higher Education & Research, Wardha, IND; 2 Emergency Medicine, Apollo Hospitals, Hyderabad, IND; 3 Ophthalmology, Heritage Institute of Medical Sciences, Varanasi, IND

**Keywords:** cardiovascular diseases, dysbiosis, gut-brain axis, gut microbiota, microbial metabolites, therapeutic strategies

## Abstract

Cardiovascular diseases (CVDs) remain the leading cause of morbidity and mortality worldwide despite advances in medical research and therapeutics. Emerging evidence suggests a significant role of the gut-brain axis, a complex communication network involving the gut microbiota, central nervous system, and cardiovascular system, in modulating cardiovascular health. The gut microbiota influences systemic inflammation, neurohumoral pathways, and metabolic processes, which are critical in the pathogenesis of CVD. Dysbiosis, or an imbalance in the gut microbiota, has been implicated in various cardiovascular conditions, including hypertension, atherosclerosis, and heart failure. This comprehensive review aims to elucidate the intricate relationship between the gut microbiome, brain, and cardiovascular system, highlighting the mechanisms by which gut-derived signals affect cardiovascular function. Key microbial metabolites, such as short-chain fatty acids (SCFAs) and trimethylamine N-oxide (TMAO), and their impact on vascular health and blood pressure regulation are discussed. Furthermore, the review explores potential therapeutic strategies targeting the gut-brain axis, including probiotics, prebiotics, dietary modifications, and pharmacological interventions, to improve cardiovascular outcomes. Despite promising findings, the field faces challenges such as individual variability in microbiome composition, complexities in gut-brain interactions, and the need for robust clinical trials to establish causality. Addressing these challenges through interdisciplinary research could pave the way for innovative, personalized therapeutic approaches. This review provides a comprehensive understanding of the gut-brain-cardiovascular axis, underscoring its potential as a novel target for preventing and treating CVD.

## Introduction and background

Cardiovascular diseases (CVDs) continue to be the leading cause of morbidity and mortality worldwide, accounting for approximately 31% of all global deaths annually [1]. These diseases encompass a broad spectrum of conditions, including coronary artery disease, hypertension, heart failure, and stroke, each contributing significantly to the overall healthcare burden [2]. Despite significant advancements in medical research, diagnosis, and therapeutic interventions, the prevalence of CVD is on the rise. An aging population, sedentary lifestyles, poor dietary habits, and the growing incidence of metabolic disorders such as diabetes and obesity drive this increase. Traditionally, the focus of cardiovascular research has been on well-established risk factors, such as hypertension, dyslipidemia, and smoking. However, in recent years, the scientific community has begun to recognize the importance of more complex systemic interconnections that influence cardiovascular health [3].

One of the most intriguing and rapidly emerging fields in this context is studying the gut-brain axis. The gut-brain axis is the bidirectional communication network between the gastrointestinal tract and the central nervous system (CNS), involving neural, hormonal, and immune pathways [4]. Within this network, the gut microbiota, a diverse community of trillions of microorganisms residing in the gastrointestinal tract, has garnered significant attention for its potential role in modulating gastrointestinal function and systemic health, including cardiovascular physiology [5]. An imbalance in the composition of the gut microbiota, known as dysbiosis, has been implicated in various systemic diseases, including metabolic syndrome, neuropsychiatric disorders, and cardiovascular diseases. The mechanisms linking the gut microbiota to cardiovascular health are multifaceted, involving microbial metabolites, systemic inflammation, and neurohumoral pathways that can influence vascular function, blood pressure regulation, and cardiac structure and function [5].

Given these emerging insights, it is becoming increasingly evident that the gut-brain axis represents a novel and promising target for understanding and potentially mitigating cardiovascular disease. Exploring this axis in the context of CVD could open new avenues for prevention and treatment, making it a critical area of research that holds the potential to reshape our understanding of cardiovascular health [6]. The primary objective of this comprehensive review is to elucidate the interconnectedness between the gut microbiome, the brain, and the cardiovascular system, focusing on understanding how disruptions in the gut-brain axis can influence cardiovascular health. This review aims to synthesize current knowledge on the mechanisms linking gut microbiota to cardiovascular function and disease, highlighting the role of microbial metabolites, neuroimmune pathways, and neural circuits mediating these effects. By delving into these complex interactions, we seek to provide a holistic perspective on the gut-brain-cardiovascular nexus.

## Review

The gut-brain axis: an overview

The gut-brain axis (GBA) is a complex, bidirectional communication network linking the gastrointestinal (GI) tract with the central nervous system (CNS). This system maintains homeostasis and influences physiological and psychological processes [7]. Anatomically, the GBA involves multiple neural pathways, including autonomic nerves that connect the brainstem and spinal cord to various abdominal organs. This intricate network facilitates localized control of GI functions and broader regulatory effects on overall health. The key components of the gut-brain axis include the gut microbiota, enteric nervous system (ENS), vagus nerve, and the CNS [8]. Communication within the GBA flows in both directions. The brain regulates gut function through neural pathways, such as the vagus nerve, hormonal signals from the hypothalamic-pituitary-adrenal (HPA) axis, and neurotransmitter release. In return, signals from the gut can influence brain function, affecting mood, cognition, and pain perception through mechanisms involving microbial metabolites and inflammatory mediators [9]. The gut microbiota produces several neuroactive compounds, including serotonin, gamma-aminobutyric acid (GABA), and melatonin, which can influence mood and behavior. Additionally, short-chain fatty acids (SCFAs) produced by gut bacteria are critical in regulating inflammation and maintaining gut health [10]. These metabolites can cross the blood-brain barrier and impact brain function. The GBA is also involved in immune signaling, where cytokines produced in response to gut microbiota can influence CNS activity. Dysbiosis, or an imbalance in gut microbiota, can lead to altered immune responses, negatively impacting gut health and neurological conditions [10]. This dynamic interplay highlights gut health's profound impact on mental well-being and vice versa, underscoring the importance of a healthy microbiome for overall health [11].

Gut microbiota and cardiovascular disease

The gut microbiota is a diverse and intricate community of microorganisms, primarily composed of bacteria, that play essential roles in maintaining human health. Key microbial species include Firmicutes, crucial for fermenting dietary fibers into short-chain fatty acids (SCFAs) that possess anti-inflammatory properties and are vital for gut health [12]. Another significant group, Bacteroidetes, aids in the degradation of complex carbohydrates, facilitating nutrient absorption. Additionally, Actinobacteria, including beneficial strains like Bifidobacterium, support immune function and contribute to maintaining a healthy gut environment. The interactions among these microbial communities influence digestion and regulate the host’s immune responses and metabolic processes, underscoring their critical role in overall health [13]. Dysbiosis is an imbalance in the gut microbiota, characterized by reduced microbial diversity and increased pathogenic species. Various mechanisms have linked this condition to several cardiovascular diseases [14]. For instance, dysbiosis can contribute to hypertension by increasing levels of pro-inflammatory cytokines and altering gut permeability, leading to systemic inflammation that elevates blood pressure. In atherosclerosis, gut microbiota changes can promote trimethylamine N-oxide (TMAO), a metabolite associated with increased cholesterol deposition in arterial walls [15]. Dysbiosis may also activate Toll-like receptors (TLRs), triggering inflammatory pathways that exacerbate plaque formation. Moreover, dysbiosis has been implicated in heart failure, as it can disrupt the gut barrier, enable bacterial translocation into the bloodstream, fuel systemic inflammation, and impair cardiac function [15]. Several metabolites produced by gut microbiota have significant implications for cardiovascular health. SCFAs, particularly butyrate, are produced through the fermentation of dietary fibers and are known to exert anti-inflammatory effects while improving endothelial function. A decrease in SCFA-producing bacteria is often observed in individuals with cardiovascular diseases, highlighting their protective role [16]. Another critical metabolite is TMAO, which gut bacteria produce from dietary choline and carnitine [17]. Elevated TMAO levels are associated with an increased risk of atherosclerosis, as they promote cholesterol accumulation in arterial walls and enhance platelet activation, contributing to thrombus formation. Lastly, bile acids, also influenced by gut microbiota, can affect lipid metabolism. Dysbiosis-induced alterations in bile acid metabolism can impair cholesterol elimination, raising plasma low-density lipoprotein (LDL) levels and contributing to atherosclerosis [17]. The impact of gut microbiota and their metabolites on cardiovascular disease is outlined in Table 1.

**Table 1:** Impact of gut microbiota and their metabolites on cardiovascular disease TMA: Trimethylamine, TMAO: Trimethylamine N-oxide, SCFAs: Short-chain fatty acids, GPCR: G-protein-coupled receptor, LPS: Lipopolysaccharides, IL: Interleukin, NF-kB: Nuclear factor kappa B, CVD: Cardiovascular disease.

Gut Microbiota	Metabolites	Cardiovascular Effects	Mechanism
Trimethylamine-producing bacteria (e.g., Lachnospiraceae, Ruminococcaceae) [18]	Trimethylamine (TMA) converted to TMAO	Increased risk of atherosclerosis and thrombosis	Promotes cholesterol deposition, impairs reverse cholesterol transport, and enhances platelet reactivity and clot formation.
Bacteroides [19]	Short-chain fatty acids (SCFAs) (e.g., acetate, butyrate, propionate)	Anti-inflammatory effects, improved endothelial function	SCFAs regulate blood pressure, reduce inflammation, and improve endothelial function through G-protein-coupled receptor (GPCR) signaling.
*Akkermansia muciniphila* [20]	Short-chain fatty acids (SCFAs) (e.g., propionate)	Improved metabolic profile, reduced risk of obesity and related CVD	Enhances mucosal barrier function, regulates lipid metabolism, and modulates inflammation.
Lactobacillus [21]	Lactic acid, SCFAs	Blood pressure regulation, improved lipid profile	It produces antihypertensive peptides, improves the gut barrier, and modulates lipid metabolism.
Prevotella [22]	Propionate	Reduced risk of hypertension, improved glucose metabolism	SCFA-mediated regulation of blood pressure and glucose homeostasis through improved insulin sensitivity.
Ruminococcus [23]	Butyrate	Anti-inflammatory effects, reduced risk of atherosclerosis	Enhances gut barrier integrity and reduces systemic inflammation through inhibition of NF-kB pathway.
*Escherichia coli* [24]	Lipopolysaccharides (LPS)	Increased risk of inflammation and atherosclerosis	LPS triggers systemic inflammation through Toll-like receptor 4 (TLR4) activation, contributing to endothelial dysfunction and atherosclerosis.
*Faecalibacterium prausnitzii* [25]	Butyrate	Anti-inflammatory effects, cardioprotective	Produces anti-inflammatory cytokines (e.g., IL-10), reduces oxidative stress, and improves gut barrier function.

The brain’s role in cardiovascular regulation

Understanding the interplay between the brain and cardiovascular system is crucial for recognizing how psychological factors influence heart health. This section focuses on two critical areas: the role of the autonomic nervous system in cardiovascular regulation and the impact of stress and depression on cardiovascular risk [26]. The autonomic nervous system (ANS) plays a vital role in regulating heart rate, blood pressure, and vascular tone. It consists of two main branches: the sympathetic nervous system (SNS) and the parasympathetic nervous system (PNS) [27]. The SNS increases heart rate and blood pressure during stress or physical activity by releasing catecholamines, which enhance cardiac output and promote vasoconstriction. In contrast, the PNS lowers heart rate and promotes relaxation via the vagus nerve, counterbalancing the effects of the SNS [27]. This balance between sympathetic and parasympathetic activity is essential for maintaining cardiovascular homeostasis. Dysregulation of the ANS can lead to various cardiovascular issues, including hypertension and arrhythmias. Chronic stress often leads to sustained sympathetic activation, resulting in increased heart rate and blood pressure, both of which contribute to the development of cardiovascular diseases. The intricate communication between the brain and heart emphasizes the importance of neurocardiology in understanding cardiovascular health [28]. Psychological factors such as stress and depression significantly impact cardiovascular health. Chronic stress activates the hypothalamic-pituitary-adrenal (HPA) axis, elevating cortisol levels. This hormonal shift can lead to insulin resistance, inflammation, and endothelial dysfunction, significant risk factors for cardiovascular disease. Moreover, stress often drives negative lifestyle behaviors, such as increased smoking or sedentary activity, which further compound cardiovascular risks [29]. Depression shares a bidirectional relationship with cardiovascular disease. Individuals with depression are at a higher risk of developing cardiovascular conditions due to factors like increased inflammation and altered autonomic regulation. Research shows that depression can accelerate the onset of cardiovascular risk factors, such as hypertension and diabetes, by about six months compared to non-depressed individuals. Furthermore, patients with existing cardiovascular diseases who also suffer from depression tend to have worse outcomes, including higher mortality rates within five years of diagnosis [30]. The roles of brain regions, neurotransmitters, and their impact on cardiovascular regulation are summarized in Table 2.

**Table 2:** Brain regions, neurotransmitters, and their roles in cardiovascular regulation GABA: Gamma-aminobutyric acid, CRH: Corticotropin-releasing hormone, PAG: Periaqueductal gray, NTS: Nucleus Tractus Solitarius, SNS: Sympathetic nervous system, PNS: Parasympathetic nervous system, ANS: Autonomic nervous system, HPA: Hypothalamic-pituitary-adrenal.

Brain Region	Neurotransmitters/Hormones	Cardiovascular Effects	Mechanism
Medulla oblongata [31]	Glutamate, GABA	Regulation of heart rate and blood pressure	Houses the cardiac and vasomotor centers that control autonomic output.
Hypothalamus [32]	Corticotropin-releasing hormone (CRH), Vasopressin	Stress-induced changes in blood pressure and heart rate, fluid balance	Integrates autonomic and endocrine responses; influences sympathetic and parasympathetic output.
Amygdala [33]	Noradrenaline, Serotonin	Modulation of heart rate variability and blood pressure during emotional stress	Activates the sympathetic nervous system during fear or anxiety, increasing heart rate and blood pressure.
Insular cortex [34]	Glutamate, GABA	Control of heart rate and blood pressure, perception of visceral sensations	Processes information related to visceral and autonomic functions, influencing cardiovascular regulation.
Cerebellum [35]	Glutamate, GABA	Coordination of cardiovascular responses during physical activity	Modulates autonomic responses to maintain cardiovascular stability during movement.
Prefrontal cortex [36]	Dopamine, Glutamate	Modulation of heart rate variability, regulation of stress response	Inhibits the amygdala and hypothalamus; involved in top-down control of autonomic function.
Periaqueductal gray (PAG) [37]	Glutamate, GABA	Regulation of heart rate blood pressure during stress and pain	Coordinates autonomic and behavioral responses to stressors and pain modulation affecting cardiovascular control.
Nucleus tractus solitarius (NTS) [38]	Glutamate, GABA	Reflex regulation of heart rate and blood pressure (e.g., baroreflex)	The primary integration center is for visceral sensory information; it modulates autonomic output via the vagus nerve.
Dorsal vagal complex [39]	Acetylcholine	Parasympathetic control of heart rate, digestion	Modulates vagal output to the heart and gastrointestinal tract, reducing heart rate and increasing digestion.

Gut-brain axis and cardiovascular disease: mechanistic insights

The gut-brain axis plays a critical role in the modulation of cardiovascular disease (CVD) through various mechanisms, including neuro-immune interactions, metabolic pathways, and neural connections. The gut microbiota significantly impacts systemic inflammation, closely tied to vascular health [40]. Dysbiosis, or an imbalance in gut microbiota, can lead to increased intestinal permeability, often called "leaky gut." This condition allows the translocation of lipopolysaccharides (LPS) and other inflammatory mediators into the bloodstream, triggering systemic inflammatory responses that exacerbate CVD risk factors such as hypertension and atherosclerosis [40]. Proinflammatory cytokines, including tumor necrosis factor-alpha (TNF-α), interleukin-1 beta (IL-1β), and IL-6, are elevated in this context, contributing to vascular inflammation and endothelial dysfunction. Additionally, gut-derived signals can influence immune responses by activating pattern recognition receptors (PRRs) on host cells, further modulating systemic inflammation and potentially leading to adverse cardiovascular outcomes [40]. The gut microbiome also interacts with metabolic pathways regulating lipid metabolism and glucose homeostasis. Microbial metabolites such as short-chain fatty acids (SCFAs) have been shown to exert anti-inflammatory effects and positively influence lipid profiles by enhancing fat oxidation and lowering cholesterol levels [41]. In contrast, the production of trimethylamine N-oxide (TMAO) from dietary precursors by gut bacteria has been linked to increased cholesterol deposition in arterial walls, promoting atherosclerosis. Moreover, gut-brain signaling pathways involving hormones like leptin and ghrelin regulate appetite and energy balance, which is crucial for maintaining metabolic health. Disruptions in these pathways can lead to obesity and insulin resistance, both of which are significant risk factors for CVD [41]. The vagus nerve is a vital conduit for communication between the gut and the brain, affecting cardiovascular function. Vagal signaling can modulate heart rate variability and has been associated with anti-inflammatory effects that protect against CVD. Activation of the vagus nerve has been shown to reduce systemic inflammation by inhibiting proinflammatory cytokine production, thereby enhancing vascular health [42]. Furthermore, dysregulation of vagal tone may contribute to the development of CVD through mechanisms involving altered gut motility and changes in gut microbiota composition. The interplay between the enteric nervous system (ENS) and the central nervous system (CNS) underscores the importance of maintaining gut health for optimal cardiovascular function [42]. Mechanistic insights into the gut-brain axis and cardiovascular disease are summarized in Table 3.

**Table 3:** Gut-brain axis and cardiovascular disease: mechanistic insights SCFAs: Short-chain fatty acids, TMAO: Trimethylamine N-oxide, PRRs: Pattern recognition receptors, TNF-α: Tumor necrosis factor-alpha, IL-1β: Interleukin-1 beta, IL-6: Interleukin-6, NF-kB: Nuclear factor kappa B, GALT: Gut-associated lymphoid tissue, 2-AG: 2-Arachidonoylglycerol.

Pathway	Key Players	Mechanism	Cardiovascular Implications
Microbial metabolites [43]	Trimethylamine N-oxide (TMAO), Short-chain fatty acids (SCFAs)	TMAO: Induces inflammation, SCFAs: Anti-inflammatory effects	TMAO: Promotes atherosclerosis and thrombosis, SCFAs: Improve endothelial function and reduce blood pressure
Vagus nerve signaling [39]	Vagal afferents	Bidirectional communication between the gut and brain	Modulates heart rate variability, reduces stress-induced cardiovascular changes
Immune system activation [44]	Gut-associated lymphoid tissue (GALT), Cytokines	Gut dysbiosis leads to systemic inflammation via cytokine release	Chronic inflammation contributes to atherosclerosis and hypertension
Autonomic nervous system [45]	Sympathetic and parasympathetic nerves	Dysbiosis affects autonomic balance, increasing sympathetic tone	Elevated sympathetic activity leads to hypertension, increased heart rate, and risk of arrhythmias
Hypothalamic-pituitary-adrenal (HPA) axis [46]	Corticotropin-releasing hormone (CRH), Glucocorticoids	Gut microbiota influence stress responses, altering HPA axis activity	A dysregulated HPA axis can lead to hypertension and increased cardiovascular risk
Intestinal permeability [47]	Tight junction proteins, Endotoxins (e.g., LPS)	Increased permeability allows endotoxins to enter the circulation	Endotoxemia contributes to systemic inflammation and endothelial dysfunction, promoting atherosclerosis
Microbial neurotransmitters [48]	Serotonin, GABA	Gut microbes produce neurotransmitters influencing brain function	Altered neurotransmitter levels affect autonomic regulation and stress responses, impacting cardiovascular health
Gut microbiota dysbiosis [49]	Decreased Bifidobacterium, increased Enterobacteriaceae	An imbalance in microbial composition leads to metabolic endotoxemia	Increased risk of metabolic syndrome, hypertension, and atherosclerosis
Endocannabinoid system [49]	Anandamide, 2-Arachidonoylglycerol (2-AG)	Modulates gut permeability, inflammation, and stress responses	Dysregulation can lead to increased cardiovascular risk, hypertension, and inflammation

Clinical implications and therapeutic potential

Modulating the gut microbiome and targeting the gut-brain axis offer significant promise for preventing and treating cardiovascular disease (CVD). Several strategies are being explored to harness these connections for clinical benefits [50]. One primary approach to modulating the gut microbiota involves probiotics, prebiotics, and dietary interventions. Probiotic supplementation with beneficial bacteria, such as Lactobacillus, Bifidobacterium, and Akkermansia, can help restore a healthy gut microbiome composition, which is often disrupted in CVD individuals. Prebiotics, found in fiber-rich foods like fruits, vegetables, whole grains, legumes, and nuts, selectively promotes the growth of beneficial gut bacteria that produce short-chain fatty acids (SCFAs) with anti-inflammatory properties [51]. Additionally, dietary interventions aimed at reducing the intake of TMAO precursors, such as red meat, eggs, and dairy, while increasing the consumption of prebiotic-rich plant foods may enhance gut microbiome composition and improve overall cardiovascular health [52]. Pharmacological approaches targeting the gut-brain axis are also under investigation. This includes using antibiotics to selectively target pathogenic bacteria that may contribute to inflammation and elevate cardiovascular risk. Additionally, psychobiotics, probiotics with potential mental health benefits, are gaining attention for their capacity to improve gut health while positively affecting emotional well-being. These interventions may help mitigate stress-related impacts on cardiovascular health; however, more research is needed to better understand their clinical efficacy and safety [53]. Lifestyle modifications also play a pivotal role in maintaining a healthy gut-brain-cardiovascular axis. A balanced diet rich in fruits, vegetables, whole grains, legumes, and nuts supports a diverse and beneficial gut microbiome, reducing inflammation and improving cardiovascular outcomes [54]. Regular physical activity has been shown to foster microbial diversity while reducing CVD risk factors such as obesity and hypertension. Moreover, effective stress management techniques, such as mindfulness meditation, yoga, and relaxation exercises, help maintain a healthy gut-brain axis by mitigating the adverse effects of stress on cardiovascular health [54]. The clinical implications and therapeutic potential of targeting the gut-brain axis in cardiovascular disease are summarized in Table 4.

**Table 4:** Clinical implications and therapeutic potential of targeting the gut-brain axis in cardiovascular disease SCFAs: Short-chain fatty acids, FMT: Fecal microbiota transplantation, DASH: Dietary Approaches to Stop Hypertension, LDL: Low-density lipoprotein, TMAO: Trimethylamine N-oxide, DMB: Dimethylbutanol, HPA: Hypothalamic-pituitary-adrenal.

Clinical Implication	Therapeutic Strategy	Mechanism of Action	Potential Cardiovascular Benefits
Microbiota-related inflammation [55]	Probiotics, Prebiotics	Modulate gut microbiota composition, reduce systemic inflammation	Decreased risk of atherosclerosis, improved endothelial function
Dysbiosis-induced hypertension [56]	Dietary interventions (e.g., DASH diet)	Restore microbiota balance, reduce blood pressure via SCFA production	Reduced blood pressure, lower risk of stroke and heart attack
Metabolic endotoxemia [57]	Antibiotics, fecal microbiota transplantation (FMT)	Reduce harmful bacteria and endotoxin levels in the gut	Decreased systemic inflammation, improved lipid profile
Vagal nerve dysfunction [58]	Vagus nerve stimulation	Enhance parasympathetic activity, reduce sympathetic tone	Improved heart rate variability, lower risk of arrhythmias
Stress and anxiety-induced cardiovascular risk [59]	Psychobiotics (e.g., *Lactobacillus rhamnosus*)	Modulate gut-brain signaling, reduce anxiety and stress responses	Improved autonomic balance, reduced blood pressure and heart rate
Gut-related metabolic syndrome [60]	Metformin, bariatric surgery	Alter gut microbiota, improve insulin sensitivity	Improved glucose metabolism, reduced risk of cardiovascular disease
Hyperlipidemia and dyslipidemia [61]	Berberine, omega-3 fatty acids	Modulate gut microbiota, reduce lipid absorption and inflammation	Lower LDL cholesterol, reduced risk of atherosclerosis
TMAO-related cardiovascular risk [62]	TMAO inhibitors (e.g., DMB), dietary choline restriction	Reduce TMAO production from dietary precursors	Decreased risk of atherosclerosis and thrombotic events
Intestinal barrier dysfunction [63]	Butyrate supplementation, probiotics	Strengthen intestinal barrier, reduce endotoxin leakage	Reduced systemic inflammation, improved cardiovascular health
HPA axis dysregulation and cardiovascular risk [64]	Stress management techniques (e.g., mindfulness, yoga)	Reduce HPA axis overactivity, lower cortisol levels	Improved blood pressure control, reduced cardiovascular stress

Challenges and controversies

Current research on the gut microbiome and its health implications, particularly cardiovascular diseases, faces several significant challenges. One of the primary issues is establishing a direct causal relationship between specific microbiome compositions and health outcomes. Many studies remain observational, making it difficult to determine whether microbiome alterations cause health issues or if pre-existing health conditions alter the microbiome [64]. The multifactorial nature of diseases further compounds the complexity, as numerous environmental, genetic, and lifestyle factors can simultaneously influence the microbiome and disease states. Additionally, the diversity of study designs, ranging from population demographics to microbiome analysis methodologies, creates inconsistencies in findings, complicating the ability to draw generalized conclusions about the microbiome's role across different populations [65]. Another significant challenge is the individual variability in microbiome composition, which presents obstacles and opportunities for personalized medicine. The gut microbiome is highly individualized and shaped by diet, genetics, environment, and overall health status [66]. This variability complicates the development of universal therapeutic interventions, as treatments effective for one individual may not yield the same results in another due to differences in baseline microbiota composition. As a result, personalized approaches are essential for effectively manipulating the microbiome to achieve desired health outcomes. This variability also complicates clinical translation, as a deep understanding of each individual’s microbiome is necessary before implementing targeted therapies that can meaningfully impact patient outcomes [66].

The microbiome manipulation also raises ethical and safety concerns that require careful consideration [67]. Ethical dilemmas include issues related to informed consent, especially given the unknown long-term effects of microbiota manipulation. Clinical trial participants must be fully informed about potential risks, many of which are not yet fully understood, so they can make well-informed decisions about participation. Equity in access to microbiome-based therapies also raises concerns; marginalized communities may face greater risks from experimental treatments if these interventions are not made widely available or if they inadvertently exacerbate existing health disparities [67]. Safety concerns are another critical area of focus, particularly concerning adverse reactions to microbiota manipulation. Rigorous safety assessments are necessary to mitigate risks associated with interventions like fecal microbiota transplantation (FMT) or engineered probiotics [68]. Comprehensive post-intervention monitoring ensures patient safety and tracks long-term health impacts. Furthermore, the possibility of unintended consequences from altering microbial communities highlights the need to consider both human health outcomes and broader ecological impacts [68]. The challenges and controversies in gut-brain axis research and its therapeutic applications for cardiovascular disease are summarized in Table 5.

**Table 5:** Challenges and controversies in gut-brain axis research and therapeutic application for cardiovascular disease CVD: Cardiovascular disease, FMT: Fecal microbiota transplantation.

Challenge/Controversy	Description	Implications	Potential Solutions
Complexity of gut microbiota composition [69]	Gut microbiota varies significantly between individuals due to genetics, diet, environment, and lifestyle	Difficulty in establishing standard treatments or interventions	Personalized medicine approaches, multi-omics studies
Causality vs. correlation [70]	Many studies show associations but do not establish direct causative links between gut dysbiosis and CVD	Uncertainty in the effectiveness of microbiota-targeted therapies	Longitudinal and interventional studies with well-defined endpoints
Variability in study results [71]	Differences in study designs, populations, and methodologies lead to inconsistent findings	Challenges in replicating results and developing universally applicable guidelines	Standardized protocols, meta-analyses to pool data
Lack of long-term clinical trials [72]	Most studies are short-term, limiting understanding of long-term safety and efficacy of interventions	Inadequate evidence for long-term benefits or risks of microbiota-targeted therapies	Long-term, large-scale clinical trials
Individual variability in response [73]	Different individuals respond variably to the same probiotic, prebiotic, or dietary intervention	Difficulty in predicting treatment outcomes and optimizing personalized interventions	Precision medicine, genetic and microbiome profiling
Ethical and safety concerns [74]	Interventions like FMT (fecal microbiota transplantation) have potential risks and ethical considerations	Regulatory and ethical challenges in the use of such therapies for cardiovascular conditions	Rigorous ethical guidelines, well-controlled studies
Influence of confounding factors [75]	Diet, medication use (e.g., antibiotics, statins), and comorbidities can significantly influence outcomes	Difficulty in isolating the effect of the gut-brain axis on cardiovascular outcomes	Careful study design, comprehensive control for confounders
Regulatory and standardization issues [76]	Lack of regulatory guidelines and standardization for probiotics and other microbiota-based interventions	Heterogeneity in product quality, dosage, and efficacy	Development of regulatory frameworks and quality control standards
Controversy over dietary supplement efficacy [77]	Conflicting evidence regarding the effectiveness of supplements like probiotics and prebiotics	Skepticism regarding the use of supplements as a reliable therapeutic option	Rigorous, placebo-controlled trials, clear communication of results
Overemphasis on gut microbiota [78]	Risk of oversimplifying complex cardiovascular diseases by focusing solely on gut microbiota	Neglect of other important factors like genetics, environment, and lifestyle	Integrative approaches combining multiple factors

Future directions

Precision medicine approaches hold great promise for optimizing cardiovascular health by tailoring interventions based on individual genetic and microbial profiles. In the context of the gut-brain-cardiovascular axis, this approach allows for personalized modulation of gut microbiota to mitigate cardiovascular disease (CVD) risk factors. By analyzing an individual’s microbiome alongside genetic data, healthcare professionals can design targeted interventions to enhance treatment efficacy and improve patient outcomes, offering a more precise, patient-centered model of care [79]. A key area of development within precision medicine is the creation of novel drugs that specifically target pathways within the gut-brain axis. Companies like Axial Therapeutics are at the forefront of this effort, focusing on gut-restricted small molecules that influence central nervous system (CNS) disorders via the gut microbiome. These drugs, which target neuroactive microbial metabolites (NMMs), have shown potential in treating conditions like autism spectrum disorder (ASD) and Parkinson’s disease by alleviating symptoms without significant systemic side effects. As research progresses, these therapies could extend to cardiovascular conditions, leveraging the gut-brain axis to treat or prevent CVD [80]. Multidisciplinary approaches combining neuroscience, cardiology, and microbiology are critical for advancing research in the gut-brain-cardiovascular axis. Collaborative efforts that integrate these fields allow for a comprehensive exploration of how gut microbiota influence cardiovascular health through mechanisms such as neuroendocrine signaling and inflammation. These integrative studies could lead to identifying novel therapeutic targets and biomarkers, ultimately improving CVD prevention strategies and treatment options [81].

## Conclusions

The intricate interplay between the gut, brain, and cardiovascular system has emerged as a promising frontier in understanding the pathophysiology of cardiovascular diseases (CVD). The gut-brain axis, through its complex network of neural, hormonal, and immune pathways, influences gastrointestinal and neurological functions and profoundly impacts cardiovascular health. Dysbiosis, or an imbalance in the gut microbiota, has been increasingly linked to various cardiovascular conditions, highlighting the significance of this axis in disease development and progression. The evidence reviewed suggests that microbial metabolites, systemic inflammation, and neurohumoral pathways serve as key mediators in the gut-brain-cardiovascular nexus, offering new insights into the multifaceted nature of CVD. Therapeutic strategies targeting the gut microbiome, such as probiotics, prebiotics, dietary modifications, and pharmacological interventions, present promising avenues for improving cardiovascular outcomes. However, despite these advancements, significant challenges remain, including the need for personalized approaches due to individual variability in microbiome composition and robust clinical trials to establish causality. As research in this field progresses, a deeper understanding of the gut-brain-cardiovascular axis could revolutionize the prevention and treatment of CVD, paving the way for innovative, holistic, and personalized healthcare strategies.
